# Relationship between emotional competence, dissociative symptoms and borderline personality disorder traits in female adolescents engaging in non-suicidal self-injury

**DOI:** 10.1186/s13034-026-01067-8

**Published:** 2026-03-22

**Authors:** Alexandra Otto, Ricarda Jacob, Irina Jarvers, Stephanie Kandsperger, Romuald Brunner

**Affiliations:** Department of Child and Adolescent Psychiatry and Psychotherapy, University of Regensburg, Universitaetsstraße 84, 93053 Regensburg, Germany

**Keywords:** Non-suicidal self-injury, Emotional competence, Intervention, BPD, Dissociation, Protective factor

## Abstract

**Background:**

Non-suicidal self-injury (NSSI) is a prevalent maladaptive coping strategy among adolescents, often associated with difficulties in emotional processing.

**Methods:**

This study investigated the relationship between emotional competence and NSSI in a clinical sample of 93 adolescent females (aged 12–21 years), 47% of whom met DSM-5 diagnostic criteria for NSSI. Dissociation and borderline personality disorder (BPD) traits were examined as potential moderators. Emotional competence and dissociation were assessed using self-report questionnaires, while BPD traits were evaluated dimensionally via a semi-structured clinical interview. Robust linear regression models were employed to examine main and interaction effects.

**Results:**

Findings revealed that higher emotional competence was generally associated with reduced lifetime NSSI. This protective effect was particularly evident in individuals with high levels of dissociation, especially when self-reported competencies in recognizing one’s own emotions and emotional expressiveness were high. However, a divergent pattern emerged in relation to BPD traits. While emotional competence was protective at low to moderate trait level, it was associated with increased NSSI at high BPD trait levels. This paradoxical effect was especially pronounced when individuals reported high competence in emotion recognition, regulation, and expressiveness.

**Conclusions:**

These findings suggest that the protective role of emotional competence in reducing NSSI may be limited or even reversed in the context of severe psychopathology. Identifying such moderating effects enhances our understanding of emotional processing in clinical populations and underscores the importance of tailored interventions. Particularly emotion-based therapeutic approaches may need to be adapted for individuals with high BPD symptoms or pronounced dissociative tendencies to improve treatment outcomes.

**Supplementary Information:**

The online version contains supplementary material available at 10.1186/s13034-026-01067-8.

## Introduction

Non-suicidal self-injury (NSSI) is defined as the deliberate and direct infliction of physical harm on oneself without suicidal intent [1]. Within the framework of emotion processing, NSSI is viewed as a maladaptive coping mechanism in response to emotionally distressing events [2]. Individuals’ perception and responses to emotional stress are shaped by past experiences and their perceived ability to cope [3, 4]. This process, influenced by cognitive development and social interactions, reflects emotional competence—the ability to navigate and regulate emotion-evoking events [4]. Emotional competence can be divided into two main components: emotion generation and emotion perception [5], and it has a fundamental protective effect on mental health and well-being [6–8].

Emotional competence includes emotion regulation processes, emotional expressivity, managing emotional reactivity, and perceiving emotions in oneself and others [9]—areas that are often considered altered in patients with NSSI [2, 10–12].

Consequently, higher emotional competence is generally associated with lower levels of NSSI, as it facilitates adaptive coping strategies and reduces reliance on NSSI as a means of emotional regulation [13–15]. This assumption is reflected in psychotherapeutic approaches such as dialectical behavior therapy, cognitive behavioral therapy, and emotion-focused therapy, which incorporate emotional competence training as a crucial component [16–18].

However, not all individuals with higher emotional competence exhibit lower levels of NSSI [19]. This suggests that additional factors may moderate the relationship between emotional competence and NSSI. One potential moderator is the presence of comorbid psychopathologies, which may weaken the protective effect of emotional competence [20].

Borderline personality disorder (BPD) is a particularly relevant comorbidity in this context [21]. It is characterized by a pervasive pattern of instability in self-image, affect, and interpersonal relationships [22], which can impair the ability to apply emotional competence effectively in distressing situations [23, 24]. As suggested by the competence-performance discrepancy framework, individuals with BPD traits may possess emotional skills but have difficulty applying them in emotionally challenging situations [25, 26], due to factors such as hyperreactivity, identity disturbance, and reduced prefrontal control [27–30]. BPD symptoms affect both internal and interpersonal emotional processes: those affected often struggle with emotion regulation strategies like reappraisal and mindfulness, and also show reduced use of social support and increased sensitivity to others’ emotions [31]. Although NSSI is a diagnostic criterion for BPD, only a significantly smaller proportion of the individuals who engage in NSSI meet the diagnostic threshold for the disorder [32–34]. Differences in study findings regarding aspects of emotional competence—particularly emotion regulation and emotional reactivity—suggest that BPD may moderate the relationship between emotional competence and NSSI. This moderating role has been observed both in individuals with elevated BPD traits [35] and in those with a formal BPD diagnosis, particularly in studies comparing individuals engaging in NSSI with and without a co-occurring BPD diagnosis [36].

Personality disorders, including BPD, often emerge in adolescence, with early trait-level indicators (e.g., emotional dysregulation, identity instability, NSSI) predicting persistent functional deficits later in life [37]. As adolescence marks a sensitive period for both personality and emotional development, early BPD traits may already interfere with emotional competence [38, 39]. Investigating this association is essential, as early disruptions could compromise adaptive functioning and increase long-term risk. Given that emotional competence is modifiable, early identification of such links offers a promising target for prevention [40].

Dissociation represents another potential moderator. It is a disconnection between a person’s thoughts, memories, feelings, actions or sense of who they are and therefore disrupts the integration of psychological functions like memory, identity, and consciousness [22, 41]. Dissociation is also frequently associated with NSSI [42] and dissociative symptoms are found to impair emotional processing, self-awareness and regulation, which may limit the effectiveness of emotional skills in individuals engaging in NSSI [43]. Recent meta-analytic findings suggest that dissociation primarily disrupts self-related emotional processes and is associated with maladaptive strategies like avoidance and rumination, but not with adaptive regulation [44].

Both BPD and dissociation have been strongly linked to NSSI and suicidal behavior, with higher levels of dissociation in individuals with BPD worsening NSSI [45, 46] and dissociation moderating the relationship between BPD features and suicidal ideation [47].

Given these considerations, the present study aims to examine the moderating roles of BPD traits and dissociation symptomatology in the relationship between emotional competence and NSSI. We hypothesize that higher levels of BPD traits will weaken the protective effect of emotional competence on NSSI across both internal (e.g., emotion regulation, emotional awareness) and interpersonal (e.g., social emotion perception, expressive skills) domains. In contrast, dissociative symptoms are expected to primarily disrupt self-related aspects of emotional competence, such as emotional awareness and regulation.

Understanding these moderating effects could lead to more targeted interventions for adolescents at risk for NSSI, ultimately improving treatment outcomes and supporting adaptive emotional development.

## Methods

### Participant recruitment

This study is based on previously collected questionnaire data from a broader experimental protocol [12]. Patients aged 13 to 18 who met the DSM-5 diagnostic criteria for NSSI, which required NSSI behavior on five or more days within the past year, were enrolled in the study [22]. Recruitment and data collection took place between August 2021 and January 2023. Clinical subjects were recruited from the Clinic of Child and Adolescent Psychiatry, Psychosomatics, and Psychotherapy at the University of Regensburg, Germany. Exclusion criteria included comorbid conditions such as autism, attention-deficit/hyperactivity disorder, acute psychotic disorders, bipolar disorders, brain-organic diseases, IQ < 80, and other acute psychiatric conditions affecting consent capacity. Healthy controls were recruited from the general population and reported no history of mental illness or psychiatric treatment history. Written informed consent was obtained from all participants and their guardians. Participants received compensation with a 25 Euro voucher. The study received approval from the University of Regensburg’s Ethics Committee (ID: 21–2177-101) and was registered in the German Clinical Trials Register (DRKS; ID: DRKS00026252).

### Psychological measurements

Psychological assessments were conducted to gather sociodemographic information and clinical characterization. Psychiatric diagnoses were initially screened using the German version of the Mini-International Neuropsychiatric Interview for Children and Adolescents (M.I.N.I. KID; Sheehan et al., 2010) and subsequently confirmed through expert evaluation involving certified child and adolescent psychiatrists. The M.I.N.I. KID is a standardized diagnostic interview designed to assess the 30 most common psychiatric disorders and their subtypes in children and adolescents according to DSM-IV and ICD-10 criteria. It is a child-appropriate adaptation of the adult M.I.N.I [48], with an average administration time of approximately 45 min in clinical populations. Validation studies have demonstrated substantial to excellent interrater and retest reliability [48, 49].

BPD traits were assessed using the German Version of the Semi-structured Clinical Interview for DSM-IV, Axis II (SCID II, 50), subsection for BPD. The interview evaluates the presence of the nine diagnostic criteria for BPD using a 4-point scale: 0 = “not present,” 1 = “subthreshold,” 2 = “present,” and? = “insufficient information.” Item scores were summed to obtain a dimensional BPD trait score. The administration time for the BPD section is approximately 15 min. Previous research indicates high reliability and validity in both adult and adolescent samples [51, 52]. According to DSM-IV guidelines, a formal BPD diagnosis requires the presence of at least five of the nine criteria. It is important to note that BPD diagnoses were not established through the SCID II interview in this study.

Rather, the SCID II BPD subsection was used to assess BPD traits dimensionally by summing item-level ratings across the BPD criteria, yielding a continuous severity score rather than a simple count of endorsed criteria.

Formal BPD diagnoses were made independently by experienced clinicians in the clinical context prior to study inclusion, as part of routine expert evaluation.

Dissociative symptoms were measured using the German version [53] of the Adolescent Dissociative Experiences Scale (A-DES, 54). The A-DES is a self-report measure consisting of 30 items rated on an 11-point Likert scale from 0 (“never”) to 10 (“always”). It captures the frequency of dissociative experiences such as depersonalization, derealization, amnesia, and identity confusion. The instrument has demonstrated excellent psychometric properties in both normative and clinical adolescent samples [55]. NSSI was assessed using the German Version of the Self-Injurious Thoughts and Behavior Interview (SITBI-G, 56). Inclusion in the study required participants to engage in NSSI behavior on five or more occasions in the past year without suicidal intent. Emotional competence was measured using the emotional competencies inventory (EKF-S). The EKF-S includes 62 items across four core subscales: [1] Recognizing one’s own emotions (RE) [2], Recognizing emotions in others (EA) [3], Regulating and controlling one’s own emotions (RC), and [4] Emotional expressiveness (EX). Responses are given on a 5-point Likert scale ranging from 1 (“does not apply at all”) to 5 (“applies fully”) [5].

### Psychometric measures

All analyses were conducted using non-parametric methods, and the Mann-Whitney U test was employed for all non-parametric comparisons. The Missing Completely at Random (MCAR) test [57] indicated random missing values for all questionnaires (EKF-S: χ²(728) = 762.31, *p* =.183; A-DES: χ² [25] = 24.91, *p* =.468; SCID: χ² [9] = 12.50, *p* =.186). In all cases, the proportion of missing data remained below 5%, and these were handled using the Expectation-Maximization (EM) algorithm [58]. Data points beyond the third standard deviation were identified as outliers and removed.

### Moderator analysis

Moderation analyses were conducted in R (Version 2022.12.0 + 353) using the rlm function with centered variables to mitigate multicollinearity. The relationship between emotional competence (EKF-S, independent variable) and NSSI Lifetime (dependent variable) was examined, with A-DES and SCID II acting as moderators. Interaction terms (e.g. EKF-S: A-DES, EKF-S: SCID II) were included to test for moderation effects. Age (in months) was included as a covariate to control for potential confounding effects. To account for potential deviations from normality in the residuals, the rlm function was selected for robust regression analysis. This approach increases accuracy and statistical power in the presence of small effects and assumption violations [59]. Homoscedasticity was evaluated with the Breusch-Pagan test, while independence of residuals was tested using the Durbin-Watson statistic. Multicollinearity among predictors was assessed using the Generalized Variance Inflation Factor (GVIF). To examine whether specific subscales of EKF-S influence NSSI, the main model was compared with alternative models in which the subscales of EKF-S (e.g., RE, EA, RC, EX) were included as independent variables instead of the overall score. These models were used to test the hypothesis that dissociative and BPD symptoms differentially impair specific facets of emotional competence, with BPD traits expected to weaken its protective effect on NSSI across both internal and interpersonal domains, and dissociative symptoms hypothesized to primarily disrupt internal, self-related aspects. Model fit was assessed through pseudo-R², mean squared error (MSE), root mean squared error (RMSE), Akaike’s Information Criterion (AIC), and Bayesian Information Criterion (BIC). Model comparisons were conducted to identify the model that best fits the data, providing insight into the specific aspects of emotional competence that may contribute to a reduction in NSSI. A significance level of 0.05 was used as the threshold for statistical significance, and all results underwent correction using the False Discovery Rate [60]. The assumptions of all models were evaluated and found to be satisfied.

## Results

### Sociodemographic and clinical characteristics

A total of 96 female participants took part in the study. Of these, 47 participants met the diagnostic criteria for NSSI. Participants with an NSSI diagnosis reported having engaged in an average of 115.86 (*SD* = 315.17) instances of NSSI throughout their lives. The first instance of NSSI occurred on average at the age of 12.40 (*SD* = 2.08) years. Participants with and without NSSI did not differ in age (*U* = 963.00 *p* =.114). Due to outliers with implausibly high lifetime NSSI frequencies, three observations were excluded from the moderator analysis, resulting in a final sample size of *N* = 93. Six participants had received a clinical diagnosis of BPD by expert clinicians as part of routine psychiatric evaluation. Sociodemographic details are presented in Table 1, clinical characteristics in Table 2.

**Table 1:** Sociodemografic and clinical characteristics

Group		NSSI group	Control group
Sample size		*n* = 44	*n* = 49
Age in years and month	*M* (*SD*)	15.93 (1.55)	16.76 (2.55)
Sex (%)	female	100	100
	male	0	0
Gender (%)	female	95.46	100
	male	4.54	0
	diverse	0	0
School type (%)	Mittelschule	13.6	0
	Realschule	40.9	20.4
	Gymnasium	36.4	36.7
	Ausbildung	2.3	4.1
	FOS/BOS	2.3	6.1
	Studium	2.3	32.7
	Others	2.3	0
ICD-10 psychiatric diagnoses (%)	F1	2.3	0
	F3	86.4	0
	F4	43.2	0
	F5	11.4	0
	F6	18.2	0
	F9	11.4	0
Multiple diagnoses (%)		61.4	0

School types refer to secondary schools following elementary school in Germany. Gymnasium: highest level of secondary school, regular duration of 8–9 years, general qualification for university entrance; FOS/BOS: tertiary school to achieve advanced technical college certificate, subject-related entrance qualification or general qualification for university entrance after visiting Realschule, duration of 2–3 years in addition to duration of Realschule; Realschule: intermediate level of secondary school, regular duration of 6 years; Mittelschule: 9 years of elementary school. Diagnosis groups refer to the ICD-10 (International Classification of Diseases, Tenth Revision). F1: Mental and behavioural disorders due to psychoactive substance use; F3: Mood [affective] disorders; F4: Neurotic, stress-related and somatoform disorders; F5: Behavioural syndromes associated with physiological disturbances and physical factors; F6: Disorders of adult personality and behaviour; F9: Behavioural and emotional disorders with onset usually occurring in childhood and adolescence. NSSI = non-suicidal self-injury

**Table 2:** Clinical characteristics

Group			NSSI group	Control group	Group differences
EKF-S	M (SD)	Total Score	2.60 (0.38)	3.66 (0.59)	U = 186.00, p <.001***
	M (SD)	RE	2.29 (0.54)	3.81 (0.74)	U = 143.00, p <.001***
	M (SD)	EA	3.55 (0.74)	3.94 (0.66)	U = 787.00, p =.008**
	M (SD)	RC	2.63 (0.52)	3.55 (0.62)	U = 307.00, p <.001***
	M (SD)	EX	1.94 (0.64)	3.33 (0.91)	U = 241.50, p <.001***
SCID II	M (SD)		10.12 (4.70)	0.37 (0.81)	U = 4.00, p <.001***
A-DES	M (SD)		4.13 (2.05)	1.12 (1.05)	U = 213.50, p <.001***

EKF-S: emotional competence questionnaire. RE: Recognizing one’s own emotions. EA: Recognizing emotions in others. RC: Regulation and control of one’s own feelings. EX: Emotional expressiveness. SCID II: Structured Clinical Interview for DSM-IV Axis II Personality Disorders, borderline personality disorder. A-DES: Adolescent Dissociative Experiences Scale. Bold values indicate statistically significant group differences. Significance levels are denoted as p < .05 (*), p < .01 (**), and p < .001 (***)

### Moderation

**Fig. 1:**
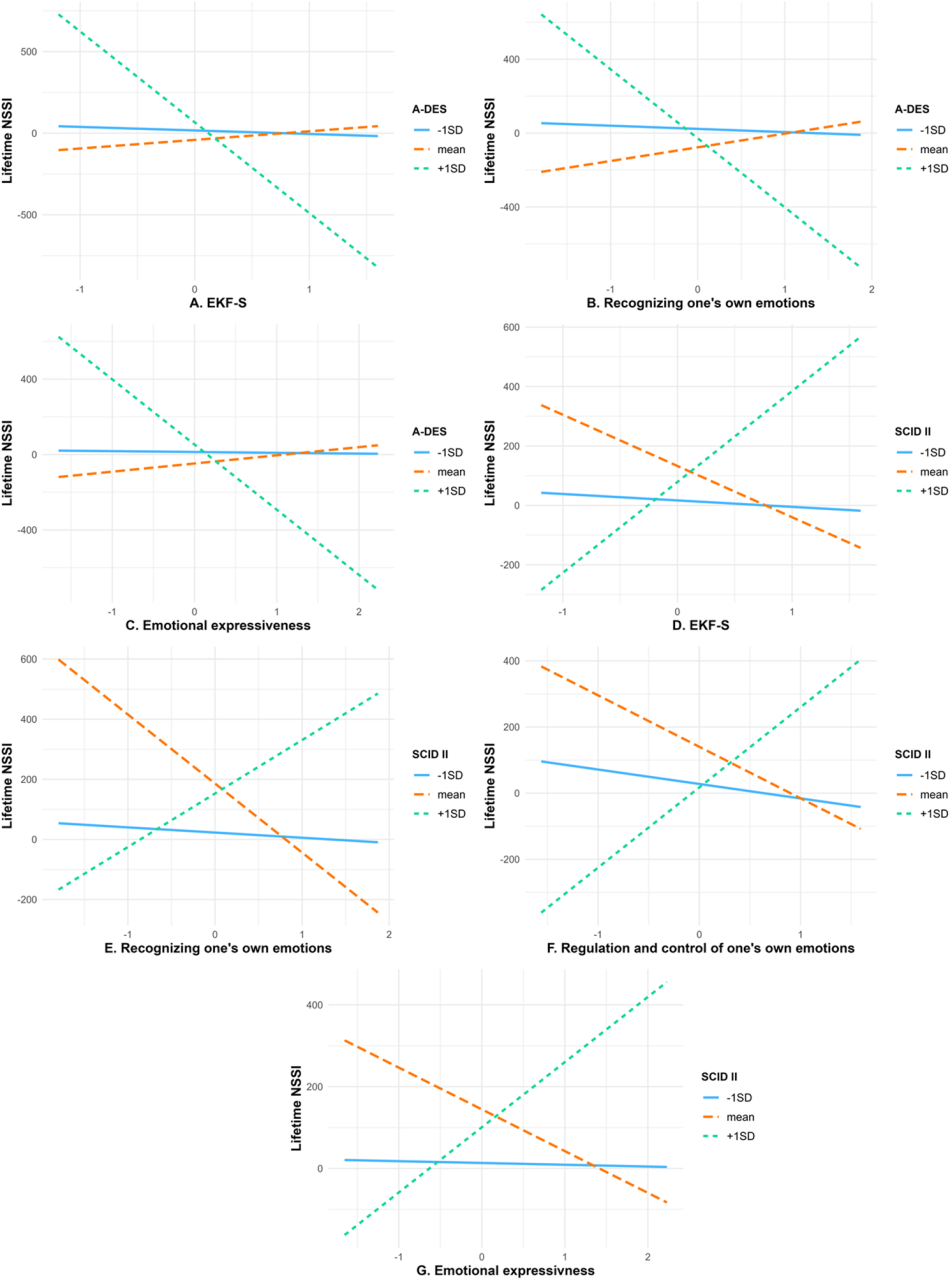
Interactions of emotional competence with dissociation and BPD predicting lifetime NSSI. **A**–**C** display the moderating effect of dissociative symptoms (A-DES) on the relationship between emotional competence and lifetime NSSI frequency, plotted at one standard deviation below the mean, the mean, and one standard deviation above the mean of dissociation. **D**–**G** illustrate the moderating effect of borderline personality disorder (BPD) traits on the association between emotional competence and lifetime NSSI, plotted at one standard deviation below the mean, the mean, and one standard deviation above the mean of BPD trait severity. A-DES = Adolescent Dissociative Experience Scale; SCID II = Semi-structured Clinical Interview for DSM-IV Personality Disorders, BPD section; EKF-S = Emotional Competence Inventory; B, C, E–G = EKF-S subscales

With the exception of age, all continuous variables were significantly correlated with one another, indicating relevant associations among emotional competence, dissociation, BPD traits, and NSSI frequency (see Table 3). The moderation analysis revealed significant main effects of emotional competence (EKF-S), dissociation (A-DES), and BPD (SCID II) on lifetime NSSI. In contrast, age did not show a significant effect. In general, higher emotional competence reduced the frequency of NSSI, while dissociation and BPD symptoms increased it. Additionally, significant interaction effects were identified between EKF-S and SCID II, as well as between EKF-S and A-DES (see Table 4 for detailed statistics). While emotional competence reduced the frequency of NSSI at high dissociation levels, the opposite effect emerges in the interaction between EKF-S and SCID II. These interaction effects are visualized in Fig. 1A and D, respectively.

**Table 3:** Bivariate correlation matrix

Measure	EKF-S	A-DES	SCID II	Age	RE	EA	EX	RC
EKF-S	1	−0.65**	−0.75**	0.26*	0.91**	0.60**	0.86**	0.84**
A-DES	−0.65**	1	0.77**	−0.11	−0.73**	−0.21*	−0.55**	−0.62**
SCID II	−0.75**	0.77**	1	−0.11	−0.80**	−0.31**	−0.65**	−0.67**
Age	0.26*	−0.11	−0.11	1	0.16	0.21*	0.25*	0.14
RE	0.91**	−0.73**	−0.80**	0.16	1	0.38**	0.77**	0.79**
EA	0.60**	−0.21*	−0.31**	0.21*	0.38**	1	0.37**	0.36**
EX	0.86**	−0.55**	−0.65**	0.25*	0.77**	0.37**	1	0.61**
RC	0.84**	−0.62**	−0.67**	0.14	0.79**	0.36**	0.61**	1

EKF-S: emotional competence inventory. RE: Recognizing one’s own emotions. EA: Recognizing emotions in others. RC: Regulation and control of one’s own feelings. EX: Emotional expressiveness. SCID II: Structured Clinical Interview for DSM-IV Axis II Personality Disorders, borderline personality disorder. A-DES: Adolescent Dissociative Experiences Scale. Correlation is significant at the 0.05 level (two-tailed) (*) or the 0.01 level (two-tailed) (**). All correlations are calculated using Spearman’s rank correlation. p-values were FDR-correctedand significant results are presented in bold

**Table 4:** EKF-S model: moderation of the effect of emotional competence on NSSI by dissociation and BPD

Variable	Estimate	Std. Error	t-value	*p*-value
(Intercept)	32.57	2.48	13.14	< 0.001***
EKF-S	−26.93	3.75	−7.19	< 0.001***
A-DES	4.03	1.34	3.01	0.003**
SCID II	5.47	0.60	9.10	< 0.001***
Age	−0.32	0.74	−0.43	0.665
EKF-S: SCID II	−3.88	0.83	−4.68	< 0.001***
EKF-S: A-DES	−5.82	1.92	−3.03	0.003**

*N* = 93, Pseudo R² = 0.13, EKF-S = Emotional Competencies Inventory, A-DES = Adolescent Dissociative Experiences Scale, SCID II = Structured Clinical Interview for DSM-IV, Axis II, subsection BPD. An FDR correction was applied. Bold values indicate statistically significant group differences. Significance levels are denoted as ***p <.001, **p <.01, *p<.05

To aid interpretation of the ± 1 SD simple-slope plots, Table S1 reports the proportion of participants falling at or beyond ± 1 SD within each group for EKF-S, A-DES, and SCID II.

To determine which specific aspects of emotional competence are moderated by psychopathology, additional moderation analyses were conducted using the subscales of the EKF-S. Higher RE scores were significantly associated with a decrease in NSSI behavior. Furthermore, significant interaction effects were found between RE and A-DES (Fig. 1B) as well as RE and SCID II (Fig. 1E). The main effects of A-DES, SCID II, and age remained unchanged (Table S2, RE Model). In contrast, no main effect was found for EA, and the interaction effects for EA x A-DES and EA x SCID II were also non-significant.

(Table S3, EA Model). Additionally, a main effect of RC was observed, showing similar effects as in the EKF-S model. Significant interaction effects were found between RC and SCID II (Fig. 1F), but not between RC and A-DES (Table S4, RC Model). Lastly, higher levels of EX were associated with a significant decrease in NSSI. The remaining main effects were identical. Significant interaction effects were found between EX and both A-DES (Fig. 1C) and SCID II (Fig. 1G) (see Table S5, EX Model, for detailed statistics). The model comparison and model fit parameters can be found in Table 5.

**Table 5:** Model fit indices for all models

Model	Pseudo *R*²	MSE	RMSE	AIC	BIC
EKF-S	0.10	93835.70	306.33	1344.71	1364.97
RE	0.09	95200.93	308.55	1346.05	1366.31
RC	0.09	95771.29	309.47	1346.61	1366.87
EA	0.08	95998.93	309.84	1346.83	1367.09
EX	0.13	91215.48	302.02	1342.07	1362.33

EKF-S = Emotional Competencies Inventory, RE = recognizing one’s own emotions, RC = regulation and control of one’s own emotions, EA = recognizing emotions in others, EX = Emotional expressiveness. Pseudo R² represents the proportion of variance explained by the model. MSE (Mean Squared Error) and RMSE (Root Mean Squared Error) indicate model prediction accuracy, with lower values reflecting better fit. AIC (Akaike Information Criterion) and BIC (Bayesian Information Criterion) assess model fit while penalizing complexity; lower values indicate better model performance. The EX Model demonstrates the best overall fit, with the highest explained variance and lowest prediction error

## Discussion

The present study investigated whether BPD traits and dissociative symptoms moderate the association between emotional competence and NSSI in a clinical sample of female adolescents. In our sample, higher emotional competence was associated with lower lifetime NSSI, suggesting a potential protective role in emotionally vulnerable youth. However, this association was not uniform: the strength and nature of the relationship varied depending on specific facets of emotional competence and psychopathology.

Among participants with dissociation scores more than one standard deviation above the mean, higher self-rated emotional competence appeared to function as a protective factor against NSSI. This association was strongest for the subscales ‘Recognizing Own Emotions’ (RE) and ‘Emotional Expressivity’ (EX), indicating that both internal and interpersonal facets of emotional competence may buffer against NSSI in the context of dissociative states.

In this context, the EX model accounted for the highest proportion of variance in NSSI (13%), although all models explained similar amounts overall. Contrary to our hypothesis that dissociation would primarily affect self-related emotional skills [41, 61], the protective role of emotional expressivity suggests that outwardly expressing emotions may help individuals with dissociative symptoms reduce their reliance on maladaptive strategies like NSSI. These findings highlight the potential value of including expressivity-focused elements in emotional competence training for this population.

A possible explanation is offered by the meta-analysis of Cavicchioli et al. [62], which found moderate to large associations between dissociation and disengagement-based emotion regulation strategies. These strategies, such as behavioral and experiential avoidance or thought suppression, may contribute to affective overmodulation [62]. In our sample, however, participants with high dissociation scores who rated their emotional awareness and emotional expressiveness as particularly high showed reduced levels of NSSI. This pattern may suggest that the ability to accurately perceive and express emotions helps counteract affective overmodulation or reduces the need for avoidance-based strategies such as NSSI. This protective effect of emotional competence on dissociative states has already been demonstrated in BPD patients with NSSI [63]. Specifically, certain aspects of emotional competence—such as high cognitive reappraisal—were found to have a protective effect against NSSI in individuals with pronounced dissociative symptoms. Consequently, individuals with NSSI and high dissociation levels might benefit particularly well from targeted emotional competence training. In the context of our study, focusing on accurately perceiving and expressing one’s own emotions seems to contribute to reduced NSSI in patients with high dissociation levels.

In contrast, a different pattern emerged concerning BPD traits. While emotional competence was associated with reduced NSSI in individuals with lower levels of BPD traits, this effect reversed at higher levels of trait severity. In this group, greater emotional competence was associated with increased NSSI, suggesting it may only be protective when BPD traits are less pronounced. This reversal was particularly evident among those who rated themselves highly in both internal domains (e.g., recognizing and regulating one’s own emotions) and external domains such as emotional expressivity.

One possible explanation is that adolescents with high levels of BPD traits may indeed be able to successfully identify, regulate, and express their emotional states [25, 26], but nonetheless continue to rely on maladaptive strategies such as NSSI as a preferred regulatory measure. This may reflect a persistent course of the disorder, in which NSSI is perceived as an established and effective means of emotional regulation, as suggested by previous studies [64, 65]. Moreover, NSSI may not only serve an intrapersonal function but also act as a form of interpersonal communication: visible signs of distress (e.g., self-inflicted wounds) can elicit support, attention, or protective responses from the social environment (Peel-Wainwright et al., 2021).

As a result, interventions focused solely on enhancing emotional competence may be insufficient for individuals with BPD, since these individuals may already perceive their current regulation strategies—including NSSI—as functional. According to Nock’s [66] functional model, NSSI can serve both intrapersonal (e.g., affect regulation, self-punishment) and interpersonal (e.g., help-seeking, influence on others) functions. In the context of BPD, these functions may reinforce the behavior, making it a persistent and perceived-effective coping strategy. For those patients, a more promising approach may involve addressing familial or systemic dynamics that contribute to the maintenance of NSSI, for example, as suggested by Bowen’s Family Systems Theory [67].

In sum, while emotional competence plays a protective role in the context of dissociative symptoms, it may become ineffective or even counterproductive in individuals with pronounced BPD traits. These contrasting effects highlight the importance of distinguishing between different psychopathological profiles when designing preventive and therapeutic interventions. Clarifying the specific function of NSSI (e.g., intrapersonal vs. interpersonal) may be particularly important for tailoring treatments that address both the skill set and its contextual use. In cases where both BPD traits and dissociative symptoms are elevated, emotional competence may have mixed or even conflicting effects. Interventions should therefore integrate emotion-focused training with stabilization techniques and interpersonal work, helping individuals distinguish adaptive from maladaptive emotional strategies and addressing both internal dysregulation and interpersonal signaling functions of NSSI.

### Strengths and limitations

This study offers several methodological strengths. It focuses on a clinically sample of adolescent females, using validated diagnostic interviews and self-report measures to ensure reliable data collection. By examining dissociative symptoms and BPD traits as moderators, the study provides a more nuanced understanding of when and for whom emotional competence protects against NSSI. Additionally, analyzing specific EKF-S subscales allows for a differentiated view of emotional competence. The use of robust regression techniques and correction for multiple comparisons further strengthens the statistical validity of the findings. However, several limitations must be acknowledged. Our cross-sectional design prevents conclusions about causality and the temporal order of emotional competence, psychopathology, and NSSI. It is possible that low emotional competence contributes to the use of NSSI as a maladaptive coping strategy. At the same time, frequent engagement in NSSI may hinder the development or maintenance of emotional skills over time—for instance, by reinforcing emotional avoidance or diminishing a sense of self-efficacy in emotion regulation. Longitudinal studies are needed to clarify these potentially bidirectional relationships and to identify developmental trajectories in high-risk adolescents. Emotional competence and dissociation were assessed via self-report, which may be influenced by bias or limited self-awareness.

An important limitation concerns the operationalization of BPD traits. In the present study, BPD traits were assessed dimensionally using the BPD subsection of the SCID II, with item-level ratings aggregated into a composite score reflecting overall trait severity. This approach provides a pragmatic severity proxy rather than a fully dimensional representation of BPD. While the SCID II captures a broad range of BPD-related features, individual criteria may differ substantially in their clinical relevance, subjective distress, and functional impact, which are not weighted differentially in the aggregated score. Further, the SCID II BPD module includes one criterion assessing NSSI and captures stress-related dissociative experiences within the BPD framework. As these aspects were examined as separate constructs in the present study, some conceptual overlap cannot be entirely ruled out. Although BPD encompasses a wide range of affective, cognitive, and interpersonal features beyond NSSI and dissociation, this overlap should be considered when interpreting associations and moderation effects involving BPD traits. Overall, the BPD composite score should be interpreted as an approximate indicator of BPD-related severity rather than a precise or fully dimensional measure of symptom expression. The exclusive focus on female participants limits the generalizability of findings to other genders. However, this sampling reflects the higher prevalence of NSSI and BPD traits typically observed in a clinical sample of adolescent females. The sample size, while adequate for main and interaction effects, may have limited power to detect complex interaction patterns. Future research should therefore employ longitudinal designs and include multi-informant or behavioral data to validate and extend these findings. Additional, the interpretation of moderation effects warrants caution. Although the statistical models tested interactions using continuous variables, graphical illustrations relied on plotting predicted values at ± 1 SD around the mean of the moderators for interpretative purposes. These visualizations are heuristic and do not imply the existence of discrete clinical subgroups. Consequently, interaction effects should be interpreted as dimensional trends rather than categorical distinctions. Lastly, results are discussed in clinically meaningful terms, the observed interaction patterns may also be influenced by methodological factors such as shared method variance inherent to retrospective self-report measures, partially restricted score distributions within the sample, and participant-related factors including response heuristics, social desirability, or cognitive dissonance when retrospectively reporting emotional skills and NSSI.

## Conclusion

This study demonstrates that emotional competence can reduce NSSI in adolescents, particularly in those with high dissociation. However, in individuals with severe BPD symptoms, higher emotional competence may be linked to increased NSSI. These findings highlight the importance of tailoring interventions to individual psychopathological profiles, as emotional competence alone may not be protective in all cases.

## Supplementary Information

Supplementary material 1 (DOCX 18 kb)

## Data Availability

The raw data supporting the findings of this study are not publicly available due to ethical restrictions and the sensitive nature of clinical data involving minors. Anonymized datasets relevant to the analyses may be made available by the corresponding author upon reasonable request.

## Abbreviations

A-DES: Adolescent dissociative experiences scale
AIC: Akaike’s information criterion
BIC: Bayesian information criterion
BPD: Borderline personality disorder
DSM-5: Diagnostic and statistical manual of mental disorders, fifth edition
DSM-IV: Diagnostic and statistical manual of mental disorders, fourth edition
EA: Recognizing emotions in others (EKF-S subscale)
RE: Recognizing one’s own emotions (EKF-S subscale)
EKF-S: Emotional competence inventory (German: Emotionale-Kompetenz-Fragebogen)
EM: Expectation–maximization (algorithm)
EX: Emotional expressiveness (EKF-S subscale)
GVIF: Generalized variance inflation factor
MCAR: Missing completely at random
M.I.N.I. KID: Mini-international neuropsychiatric interview for children and adolescents
MSE: Mean squared error
NSSI: Non-suicidal self-injury
ORCID iD: Open researcher and contributor ID
RC: Regulating and controlling one’s own emotions
RMSE: Root mean squared error
SCID II: Structured clinical interview for DSM-IV axis II disorders (BPD subsection used dimensionally)
SITBI-G: Self-injurious thoughts and behaviors interview – German version
