# Supplementary material for: Relationship between emotional competence, dissociative symptoms and borderline personality disorder traits in female adolescents engaging in non-suicidal self-injury
Source: Child Adolesc Psychiatry Ment Health. 2026 Mar 22;20:52. doi: 10.1186/s13034-026-01067-8 (PMC13063858; doi:10.1186/s13034-026-01067-8)
Supplement: Supplementary file 1 — Supplementary material 1 (DOCX 18 kb) [file 13034_2026_1067_MOESM1_ESM.docx]

| **Variable** | **Group** | **n** | **M** | **SD** | **n ≤ −1 SD (%)** | **n ≥ +1 SD (%)** |
| --- | --- | --- | --- | --- | --- | --- |
| EKF-S | Control group | 49 | 3.66 | 0.59 | 5 (10.2%) | 9 (18.4%) |
|  | NSSI group | 47 | 2.60 | 0.39 | 8 (17.0%) | 9 (19.1%) |
| A-DES | Control group | 49 | 1.12 | 1.05 | 1 (2.0%) | 10 (20.4%) |
|  | NSSI group | 47 | 4.13 | 2.05 | 10 (21.3%) | 8 (17.0%) |
| SCID II | Control group | 49 | 0.37 | 0.81 | 0 (0.0%) | 5 (10.2%) |
|  | NSSI group | 47 | 10.12 | 4.70 | 9 (19.1%) | 11 (23.4%) |

**Table S1**

**Note.** EKF-S = Emotional Competence Questionnaire; A-DES = Adolescent Dissociative Experiences Scale; SCID II = Structured Clinical Interview for DSM-IV Axis II Disorders (BPD subsection, dimensional score). Values indicate the number and percentage of participants falling at least one standard deviation below or above the group mean. Percentages are calculated within each group.

**Table S2**

*RE Model: Moderating the Effect of RE on Lifetime NSSI by A-DES and SCID II*

| **Variable** | **Estimate** | **Std. Error** | **t-value** | **p-value** |
| --- | --- | --- | --- | --- |
| (Intercept) | 31.397 | 2.563 | 12.250 | **<.001** |
| RE | -16.173 | 2.915 | -5.548 | **<.001** |
| A-DES | 4.743 | 1.391 | 3.409 | **.001** |
| SCID II | 4.893 | 0.651 | 7.520 | **<.001** |
| age | -0.488 | 0.708 | -0.689 | .493 |
| RE:SCID II | -2.031 | 0.606 | -3.353 | **.001** |
| RE:A-DES | -4.104 | 1.331 | -3.083 | **.003** |

*Note.* *N* = 93, Pseudo R² = 0.09, RE: Recognizing one’s own emotions (EKF-S subscale), A-DES = Adolescent Dissociative Experiences Scale, SCID II = Structured Clinical Interview for DSM-IV, Axis II, subsection BPD. An FDR correction was applied.

**Table S3**

*EA Model: Moderating the Effect of EA on Lifetime NSSI by A-Des and SCID II*

| **Variable** | **Estimate** | **Std. Error** | **t-value** | **p-value** |
| --- | --- | --- | --- | --- |
| (Intercept) | 44.624 | 3.017 | 14.790 | **< .001** |
| EA | -3.126 | 4.397 | -0.711 | .559 |
| A-DES | 7.413 | 2.269 | 3.267 | **.005** |
| SCID II | 6.364 | 0.829 | 7.673 | **< .001** |
| age | -1.228 | 1.387 | -0.885 | .531 |
| EA:SCID II | 0.652 | 1.128 | 0.578 | .565 |
| EA:A-DES | -4.390 | 3.278 | -1.339 | .332 |

*Note.* *N* = 93, Pseudo R² = 0.08, EA = Recognizing emotions in others (EKF-S subscale), A-DES = Adolescent Dissociative Experiences Scale, SCID II = Structured Clinical Interview for DSM-IV, Axis II, subsection BPD. An FDR correction was applied.

**Table S4**

*RC Model: Moderating the Effect of RC on Lifetime NSSI by A-Des and SCID II*

| **Variable** | **Estimate** | **Std. Error** | **t-value** | **p-value** |
| --- | --- | --- | --- | --- |
| (Intercept) | 37.165 | 2.816 | 13.198 | **< .001** |
| RC | -14.834 | 4.117 | -3.603 | **.002** |
| A-DES | 4.828 | 1.980 | 2.438 | **.030** |
| SCID II | 6.148 | 0.804 | 7.644 | **< .001** |
| age | -0.630 | 1.084 | -0.582 | .562 |
| RC:SCID II | -2.558 | 1.109 | -2.308 | **.023** |
| RC:A-DES | -2.774 | 2.841 | -0.976 | .378 |

*Note.* *N* = 93, Pseudo R² = 0.09, RC = regulation and control of one's own emotions (EKF-S subscale), A-DES = Adolescent Dissociative Experiences Scale, SCID II = Structured Clinical Interview for DSM-IV, Axis II, subsection BPD. An FDR correction was applied.

**Table S5**

*EX Model: Moderating the Effect of EX on Lifetime NSSI by A-Des and SCID II*

| **Variable** | **Estimate** | **Std. Error** | **t-value** | **p-value** |
| --- | --- | --- | --- | --- |
| (Intercept) | 32.961 | 2.365 | 13.934 | **.007** |
| EX | -25.332 | 2.333 | -10.857 | **.014** |
| A-DES | 5.453 | 1.477 | 3.691 | **.021** |
| SCID II | 4.790 | 0.574 | 8.345 | **.029** |
| age | -0.443 | 0.853 | -0.519 | .605 |
| EX:SCID II | -3.335 | 0.592 | -5.636 | **.036** |
| EX:A-DES | -5.642 | 1.361 | -4.144 | **.043** |

*Note.* *N* = 93, Pseudo R² = 0.13, EX = Emotional expressiveness (EKF-S subscale), A-DES = Adolescent Dissociative Experiences Scale, SCID II = Structured Clinical Interview for DSM-IV, Axis II, subsection BPD. An FDR correction was applied.
